# Visualization of SARS-CoV-2 particles in naso/oropharyngeal swabs by thin section electron microscopy

**DOI:** 10.1186/s12985-023-01981-9

**Published:** 2023-02-06

**Authors:** Michael Laue, Tobias Hoffmann, Janine Michel, Andreas Nitsche

**Affiliations:** 1 Advanced Light and Electron Microscopy, Center for Biological Threats and Special Pathogens (ZBS 4), Robert Koch Institute, Seestr. 10, 13353 Berlin, Germany; 2 Highly Pathogenic Viruses, Center for Biological Threats and Special Pathogens (ZBS 1), Robert Koch Institute, Seestr. 10, 13353 Berlin, Germany

**Keywords:** SARS-CoV-2, Swab sample, Thin section electron microscopy, Ultrastructure, Virus particle

## Abstract

**Background:**

SARS-CoV-2 replicates efficiently in the upper airways of humans and produces high loads of virus RNA and, at least in the initial phase after infection, many infectious virus particles. Studying virus ultrastructure, such as particle integrity or presence of spike proteins, and effects on their host cells in patient samples is important to understand the pathogenicity of SARS-CoV-2.

**Methods:**

Suspensions from swab samples with a high load of virus RNA (Ct < 20) were sedimented by desktop ultracentrifugation and prepared for thin section electron microscopy using a novel method which is described in detail. Embedding was performed in Epon or in LR White resin using standard or rapid protocols. Thin sections were examined using transmission electron microscopy.

**Results:**

Virus particles could be regularly detected in the extracellular space, embedded in a background of heterogenous material (e.g. vesicles and needle-like crystals), and within ciliated cells. Morphology (i.e. shape, size, spike density) of virus particles in the swab samples was very similar to particle morphology in cell culture. However, in some of the samples the virus particles hardly revealed spikes. Infected ciliated cells occasionally showed replication organelles, such as double-membrane vesicles. The most common cells in all samples were keratinocytes from the mucosa and bacteria.

**Conclusions:**

The new method allows the ultrastructural visualization and analysis of coronavirus particles and of infected host cells from easy to collect naso/oropharyngeal patient swab samples.

**Supplementary Information:**

The online version contains supplementary material available at 10.1186/s12985-023-01981-9.

## Background

The pandemic Severe acute respiratory syndrome coronavirus type 2 (SARS-CoV-2) primarily infects the respiratory tract [1]. In most cases the infection is restricted to the upper airways (nasal or throat epithelium) but it also can affect the lungs with severe consequences, such as pneumonia and considerable damage of the alveolar epithelial barrier in a fraction of the patients [2]. Infection with SARS-CoV-2 is confirmed by the detection of virus RNA in naso- and oropharyngeal swabs using quantitative real-time polymerase-chain reaction (qRT-PCR) [3]. Presence of viral RNA correlates with disease symptoms and cultivation/isolation of SARS-CoV-2 from these swabs [4]. Electron microscopy (EM) could demonstrate the virus in cell cultures treated with suspensions from broncho-alveolar lavage and thereby confirmed the identity of the virus as a coronavirus [5]. EM serves as an important control to demonstrate the presence of intact virus particles, because it is the only method which is able to demonstrate the virus particle (i.e. the infectious unit) itself and not only their molecular constituents (nucleic acids and protein) that could be present independently from an intact virus particle. Therefore, EM is essential in the histopathological analysis of infectious diseases caused by small pathogens, such as viruses, which are usually detected by antibodies or nuclear probes directed against distinct molecular targets of the pathogens. However, demonstration and analysis of SARS-CoV-2 particles by EM in patient samples is difficult as pointed out by a number of authors [6–8], and succeeded convincingly only in a few publications [9–12] (see [13] for a detailed review of the literature). Autopsy samples, which were, in contrast to biopsy samples, available in higher numbers, usually suffer from structural impairment by autolysis, due to the time interval between death and tissue conservation by fixation, which still may allow detection of viruses but interfere with the analysis of the ultrastructural pathology (e.g. the identification of affected cell types [7, 14]). Moreover, the virus most likely has been cleared already in many patients developing a fatal pneumonia, which would explain the difficulties to find virus particles in autopsy samples [11].

The upper airways are the best target for an analysis of the virus and the histopathology of the earlier phases of the COVID-19 disease because of the high virus RNA loads that can be measured. Biopsies would be suitable samples for studying such infections (e.g. [15]). However, biopsies are not medically indicated and are therefore not available for an investigation by EM. The most direct and minimal invasive way to get virus including, perhaps, infected cell samples from patients is to use the naso/oropharyngeal swabs obtained for qRT-PCR. Recently, virus structures at the surface of cells dried from swab suspensions could be demonstrated by scanning EM [16] but they lack the resolution which is necessary for a detailed analysis of particle ultrastructure and ultrastructural pathology of infection. Therefore, we applied a so far unpublished enrichment method for small volumes of virus suspensions to find SARS-CoV-2 in naso/oropharyngeal swab samples by thin section EM. The method involves sedimentation of a small volume (< = 100 µl) of swab suspension with an airfuge ultracentrifuge and resin embedding of the tiny pellet for thin sectioning. We provide a detailed protocol of the procedure, including a step-by-step embedding protocol, and demonstrate SARS-CoV-2 particles and infected cells.

## Material and methods

Naso- and/or oropharyngeal swabs were taken from individuals by different operators using various standard swab collection systems (with and without transport medium). Dry swab brushes were transferred into 1 ml of phosphate-buffered saline (PBS). RNA extraction and qRT-PCR were conducted as described previously [17]. Determination of the virus variant was performed using validated mutation specific PCRs for SNP N501Y, deletion H69/V70 (unpublished), PCRs of the TaqMan SARS-CoV-2 mutation panel from Thermo Fisher Scientific, or by sequencing [18].

**Fig. 1:**
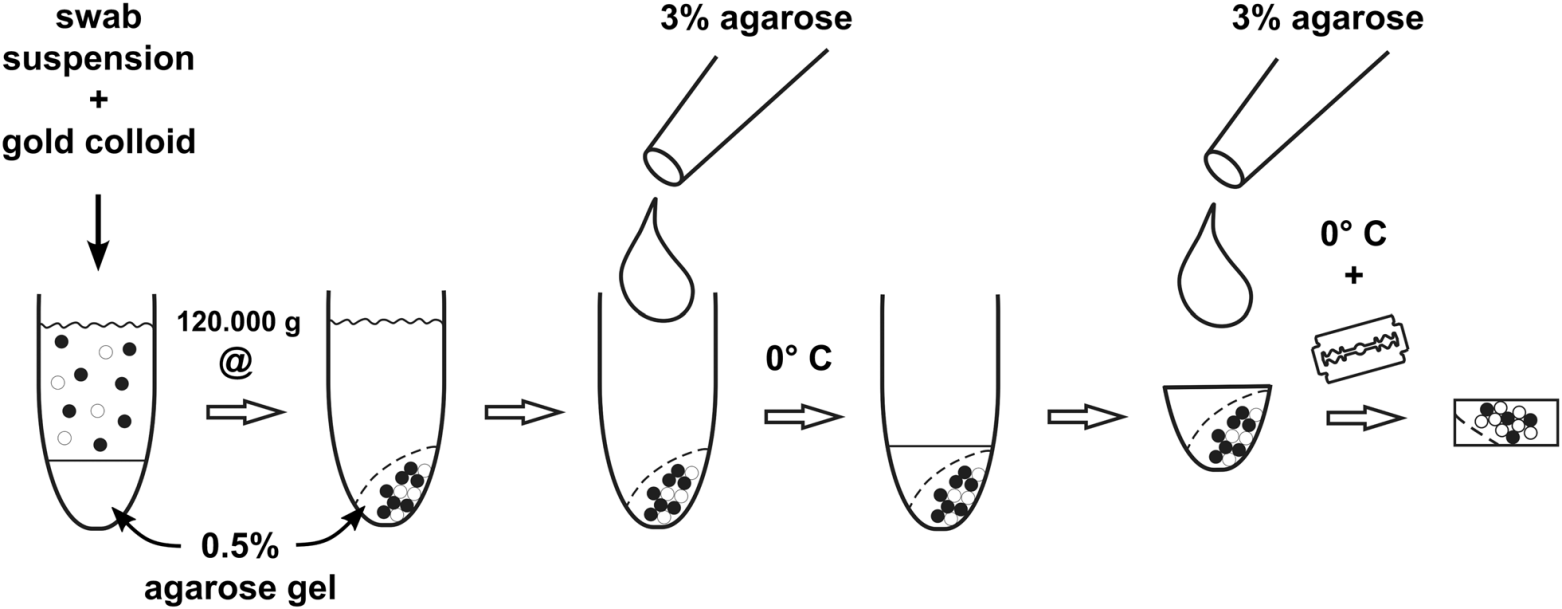
Schematic representation of particle sedimentation by airfuge ultracentrifugation and preparation of the sediment for embedding in resin

For thin section EM, we randomly selected nine samples with Ct values below 20 (a Ct value of 20.4 corresponds to 2 × 10^7^ RNA copies/ml [19]), and two samples, which were negative by PCR (see Table 1). To concentrate the particles of the suspensions, we used desktop ultracentrifugation with an airfuge (Beckman Coulter) (see Fig. 1 for a graphic overview of the procedure). The sample suspensions (90 µl) were loaded on top of a cushion (25 µl) of low-melting point agarose (0.5% in water), which was filled and solidified in a standard airfuge vial (Beckman Coulter, Part. No. 342630). The suspensions were supplemented with 10 µl of glutaraldehyde (2.5% in 0.05 HEPES buffer, pH 7.2) and 20 µl of a gold colloid suspension (15 nm cationic gold; British Biocell) to further stabilize the biological particles and to stain the pellet by the red color of the concentrated gold colloid. Centrifugation was conducted for 10 min at room temperature and approx. 120.000×*g* (20 psi) using the fixed-angle A-100/18 rotor (Beckman Coulter, Part. No. 347593). After centrifugation, the supernatant was removed and the airfuge vial was filled with 100 µl of warm (approx. 40 °C) 3% low-melting point agarose, which was solidified on ice. The hardened agarose bloc was removed from the vial with a small spatula and the red pellet was covered by a drop of 3% low-melting point agarose to avoid dispersal during further preparation steps (see Additional file 1). Embedding for thin section electron microscopy was either performed according to a standard protocol, which involved post-fixation with osmium tetroxide, tannic acid and uranyl acetate before dehydration and embedding in Epon resin ([20]; see Additional file 2 for a step-by-step protocol), or by using rapid embedding in LR White after post-fixation with 1% osmium tetroxide for 30 min at room temperature ([21]; see Additional file 3 for a step-by-step protocol). Ultrathin sections were produced at a thickness between 60 and 70 nm using a diamond knife and an ultramicrotome (UC7, Leica Microsystems). Sections were collected on naked copper grids (300 or 300 × 75 mesh), stained with uranyl acetate followed by lead citrate and coated with carbon.

**Table 1:** List of naso-/oropharyngeal swabs tested

Sample No	PCR [Ct value)	Virus variant	Remarks
1	13.53	Alpha	Frozen
2	13.73	Delta	Frozen
3	18.06	Delta	Frozen
4	16.00	Delta	Frozen
5	17.40	Delta	Frozen
6	18.60	Delta	Non-frozen
7	14.30	Delta	Non-frozen
8	17.10	Delta	Non-frozen
9	16.60	Omicron	Frozen
10	Negative	-	Frozen
11	Negative	-	Frozen

Frozen = -80 °C

Non-frozen = storage at room or refrigerator temperature

Determination of the detection limit of the entire procedure was done using suspensions of *Vaccinia virus* at defined concentration (10^6^ and 10^7^ virus particles/ml). Virus propagation in cell culture and determination of particle concentration was done as described before [22]. Airfuge sedimentation was performed as described above, except that 100 µl of the sample suspension was used. The pellets were post-fixed and embedded in LR White as described above. Ultrathin sections (60–80 nm thickness) were collected on formvar-filmed slot grids and stained with a mixture of methyl cellulose and uranyl acetate [23].

Transmission EM of the sections was performed at 120 kV (Tecnai Spirit, Thermo Fisher) and images were recorded with a side-mounted CMOS camera (Phurona, EMSIS). Electron tomography was performed at 120 kV using a bottom mounted CCD camera (Eagle 4 k, Thermo Fisher) operated at 2048 by 2048 pixels and the Tecnai tomography software (Xplore 3D, version 2.4.2, Thermo Fisher Scientific). Reconstruction was done with the Inspect3D software (version 3.0, Thermo Fisher Scientific) using the “Simultaneous Iterative Reconstruction Technique “ (SIRT) algorithm (see [24] for a step-by-step description of the reconstruction).

## Results

**Fig. 2:**
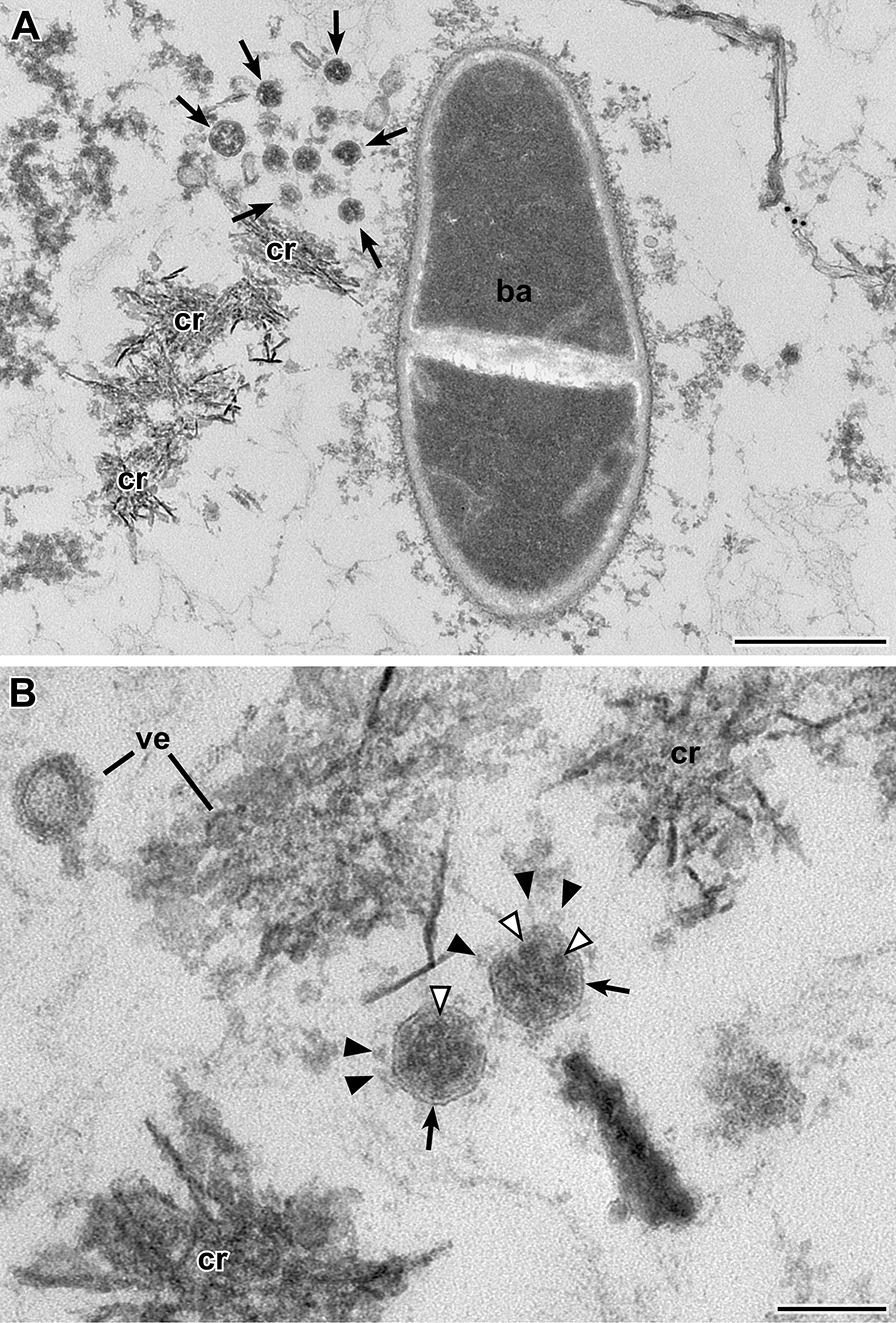
Transmission EM of a thin section through the sediment of swab sample 1 (Epon embedding). SARS-CoV-2 particles (*arrows*) are visible close to a rod-like bacterium (*ba*; **A**) and unidentified crystalline (*cr*) and vesicular (*ve*) aggregates (**A, B**). The virus particles (*arrows*; **B**) reveal all features of coronaviruses, such as an enveloping bio-membrane (*arrow*), spikes with a knobby head (*black arrowhead*), and an electron-dense, granular interior (*white arrowhead*) representing the ribonucleoprotein of the virus [8]. Note, that the granular profiles are less distinct and larger in size than reported for other preparations [8]. Scale bar in A = 0.5 µm and in B = 100 nm

**Fig. 3:**
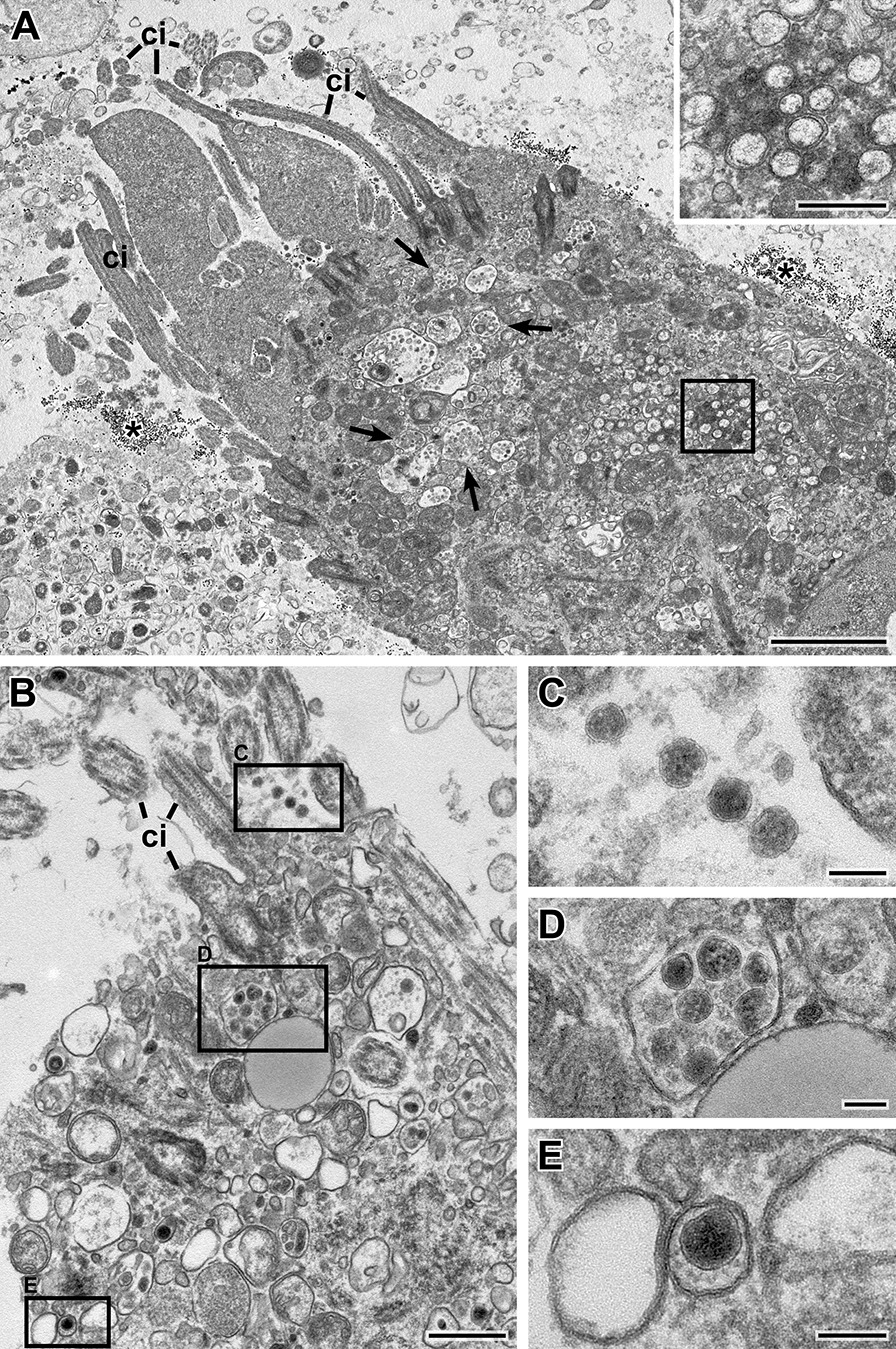
Transmission EM of a thin section through the sediment of swab sample 7 (Epon embedding). **A** Overview of ciliated cell which shows several cilia (*ci*) in this profile and which is surrounded by vesicular material and gold colloid (*). Membrane-bound inclusions (*arrows*) with virus particles and double-membrane vesicles (DMVs; *rectangle*) are visible in the cytoplasm. The inset shows a magnified view of the cytoplasmic region marked with the rectangle which contain a group of DMVs (See also Additional file 7: Fig S4B). **B-E** Another ciliated cell than in **A** which shows virus particles at the surface (**C**) and in membrane-bound compartments (**D, E**). Note that the overall spike visibility in this sample is sufficient. Scale bar in A = 2 µm, inset and B = 0.5 µm, C-E = 100 nm

We selected nine naso/oropharyngeal swab samples from different persons which revealed Ct-values below 20 by RT-PCR and two swab samples that were RT-PCR-negative (Table 1) and concentrated the sample suspension by airfuge ultracentrifugation. The resulting tiny pellet was recovered and processed for conventional thin section electron microscopy. Embedded pellets were sectioned, contrasted and examined by transmission electron microscopy. We found coronavirus particles in the first or second level of sectioning in all of the nine samples that were positive by RT-PCR but not in sections of the two samples that were negative by RT-PCR. Coronavirus particles were intermingled between bacteria and unidentified structures of various morphologies, e.g. vesicular and crystalline (Fig. 2, Additional file 4: Fig. S1), or in and around eukaryotic cells (Fig. 3, Additional file 5: Fig. S2). The virus particle profiles revealed the characteristic features of coronaviruses [8], such as a size between 60 and 140 nm (without spikes), a limiting bio-membrane, a granular interior and club-shaped spikes (Figs. 2B, 3C-E, Additional file 4: Fig. S1 and Additional file 5: Fig. S2) which allowed distinguishing them from other profiles present in the samples (see Additional file 6: Fig. S3 for a comparison of virus particle profiles and other profiles found in a PCR-negative sample). However, the fraction of particles demonstrating the entire set of characteristic features was variable among the samples. In some of the samples, spikes were visible only rarely and distinct virus particle identification was only possible if the particles were localized at high number in membrane-bound compartments of cells [25]. Remarkably, samples which are demonstrating virus particles without or only few visible spikes were found among frozen and fresh samples. In addition, the virus particle interior often appeared very dense, in some cases entirely dark (Fig. 3C–E), which impairs with the detection of virus particles if spikes are not present. Electron tomography improved the z-resolution and revealed interior substructure and spikes much better than standard imaging of the thin sections (Additional file 7: Fig. S4).

The size of coronavirus particles and their spike number measured in swab samples, in which the virus particle profiles generally showed spikes, were similar to the size and spike number measured in Vero cell culture (Table 2; see [24] for details of the Vero cell culture infection experiments and virus particle measurements using sections at 45 nm thickness). However, it is notable that the virus particles of the two measured swab samples showed a slightly smaller spike number per profile than the respective virus variant in cell culture. Moreover, the virus particles of swab sample 7 demonstrated a smaller shape factor “roundness”, along with a higher variability of the size, than the particles measured in the other samples which indicates a deviation from round/oval shape of the virus particles in sample 7. Virus particles in the swab samples sometimes appear compressed or irregular in shape. Such a systematic deviation from the usual round/oval shape of the particles most probably was responsible for the small difference in size (maximum diameter) measured for the virus particles of swab sample 7 in comparison to the virus particles of the same virus variant in cell culture, which revealed a rounder shape (Table 2).

**Table 2:** Virus particles size, shape and spike number

Sample ID	Virus variant	Maximal diameter [nm]	Perimeter [nm]	Shape factor "roundness"	Spike number per profile	Virus particles measured
Swab sample 1	Alpha	92 (6)	283 (16)	0.93 (0.04)	8 (5)	100
Cell culture	Alpha	93 (6)	284 (14)	0.94 (0.05)	10 (4)	153
Swab sample 7	Delta	96 (14)	287 (28)	0.85 (0.13)	9 (5)	100
Cell culture	Delta	92 (6)	281 (18)	0.92 (0.05)	10 (4)	153

Measurements are represented as their median with inter quartile range in brackets

The composition of the swab samples regarding bacteria and eukaryotic cells varied considerably among the swab samples studied, regardless of their virus content. Bacteria were found in all samples (see Fig. 2, Additional file 4: Fig. S1 and Additional file 5: Fig. S2), while coccoid bacteria were more frequently found than rod-like bacteria. Keratinocytes from the epidermal regions of the naso/oropharyngeal cavities were prominent in all samples studied (Additional file 4: Fig. S1, Additional file 5: Fig. S2, and Additional file 6: Fig. S3). Ciliated cells were only detected in some of the samples (Fig. 3, Additional file 8: Fig. S5A) and occasionally some of them were infected and demonstrated coronavirus particles within membrane-bound compartments of the cytoplasm (Fig. 3). Moreover, in few of the infected ciliated cells, typical double-membrane vesicles (DMVs) could be detected (Fig. 3A, Additional file 7: Fig. S4B), which indicate virus replication in the respective cell [26]. It is of note that some of the cells appeared relatively intact and only slightly structurally affected by the entire sampling and preparation procedures (Additional file 8: Fig. S5). However, while bacteria and keratinocytes were sufficiently preserved in all samples regardless of the storage conditions, other cells reveal significant damage (e.g. perforated bio-membranes) in frozen samples (Additional file 9: Fig. S6).

**Fig. 4:**
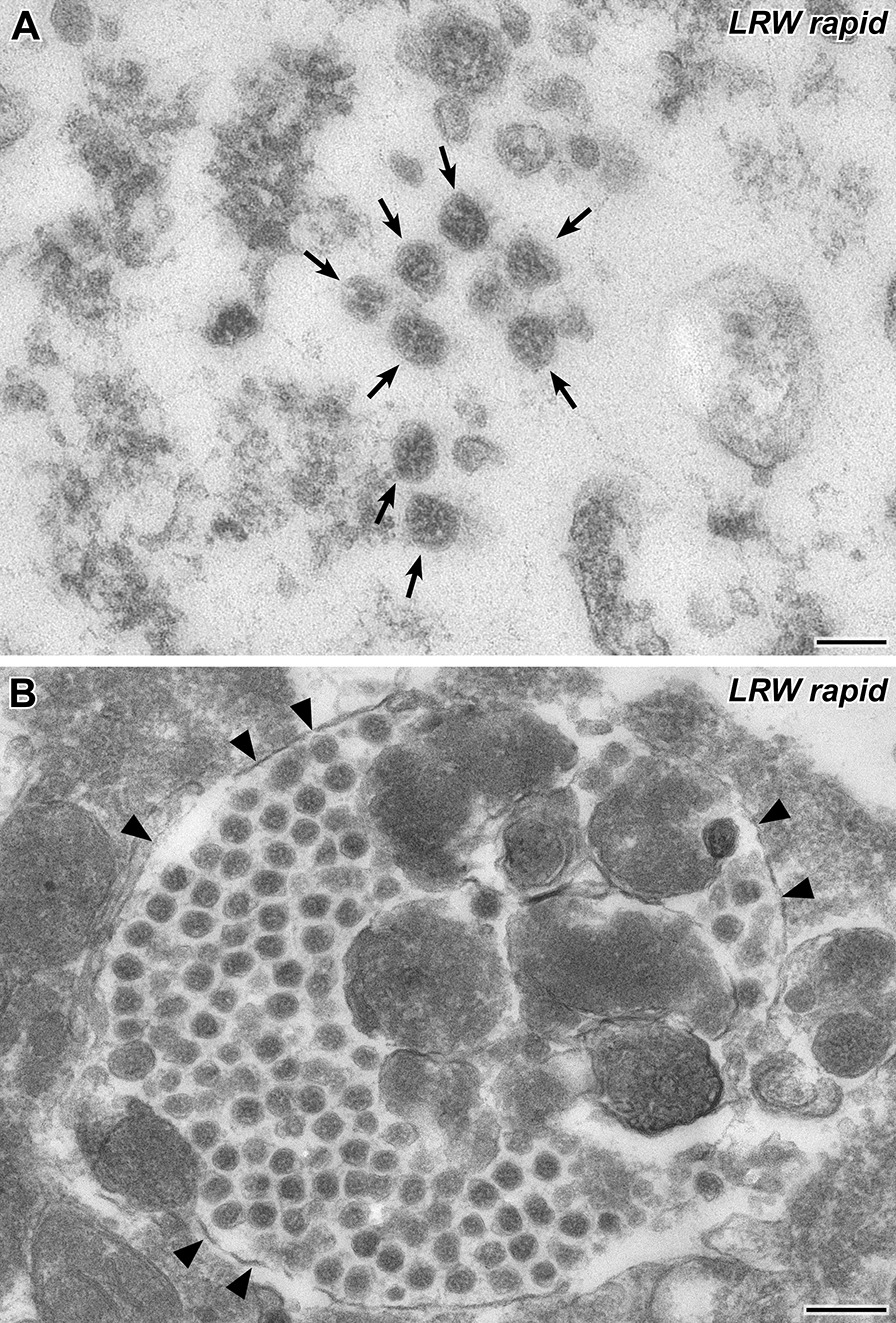
Transmission EM of a thin section through the sediment of swab sample 7 (LR White rapid embedding). **A** A group of SARS-CoV-2 particles (*arrows*) in the extracellular space. Spike visibility is insufficient (compare also spike visibility of the same sample embedded in Epon resin, Fig. 3 B-E). **B** A cytoplasmatic membrane-bound inclusion (*arrowheads*) which is densely filled with coronavirus particles. Scale bar in A = 100 nm, in B = 200 nm

To investigate whether rapid embedding could be used to investigate the swab samples or not, we post-fixed airfuge sediments of three swab samples with 1% osmium tetroxide (30 min) and embedded them in LR White resin [21] (the polymerization time was adjusted to 1 h). The entire embedding procedure lasted about 2 h. Coronavirus particles were found in all samples embedded in LR White. However, virus particles were much more difficult to identify than in the samples embedded in Epon resin, because the spikes were hardly visible in most of the virus particles (Fig. 4A). And this was specific for the LR White protocol used because swab sample 7 was embedded in both resins, Epon and LR White, and clearly revealed spikes only after Epon embedding (compare Fig. 3 with Fig. 4). A reliable identification of coronavirus particles in the LR White embedded samples was only possible if the particles were found in cells in which they typically aggregated in membrane-bound compartments of the cytoplasm (Fig. 4B; [25]).

We selected swab samples which most likely contained a high concentration of virus particles according to the RT-PCR measurements of the virus RNA load. To get an idea about the detection limit of our airfuge sedimentation method, we sedimented *Vaccinia virus* suspension at two defined concentrations (10^6^ and 10^7^ particles/ml; see [22] for a description of the determination of the actual particle concentration) and embedded the pellets in LR White (Additional file 10: Fig. S7). With a suspension of 10^7^ particles/ml, virus particles could be found in the first section through each of the pellets (3 of 3 samples), while with a suspension of 10^6^ particles/ml only part of the pellets showed virus in the first Section (6 of 8 samples).

## Discussion

Concentration of particles from naso-/oropharyngeal swabs by airfuge ultracentrifugation allowed the visualization of SARS-CoV-2 particles by thin section EM. Airfuge centrifugation in electron microscopy was mainly used in conjunction with negative staining of particles to increase particle number adsorbed at the sample carriers [27, 28]. Sample concentration of virus particles for thin section EM was usually performed by using larger ultracentrifuges which allow the processing of larger volumes (i.e. several tens of milliliters) from cell culture and the usage of density gradients for purification (e.g. [29]). Our new method concentrates all particles from a small volume of suspension obtained from a naso-/oropharyngeal swab sample within a comparatively short time (approximately 20 min) and thereby making them accessible for thin section electron microscopy.

Virus particles could be detected in the extracellular space and within ciliated cells, which is the main target cell of the virus in the upper airways [9, 10]. Remarkably, infected cells occasionally revealed replication organelles, such as double-membrane vesicles (DMVs), which are typical for coronavirus infected cell cultures [26]. Recently, DMVs were also demonstrated in infected alveolar cells from autopsy tissue [12].

The swab samples contained other cells and material besides virus particles and ciliated cells, mainly keratinocytes from the upper layers of the epidermis and, mostly coccoid, bacteria. Vesicular and needle-like crystal aggregates formed the background matrix, which most probably derived from cells or the swab material, respectively. The preservation of the keratinocytes, bacteria, viruses and background material was similar in frozen and non-frozen samples, while other cells, in particular ciliated cells, appeared extracted in the swab samples that were frozen. Thus, for optimal preservation of cells, especially if the cell biology of the virus is of interest, fresh swab samples should be considered. For diagnostic purposes, even frozen material is suitable.

The coronavirus morphology seemed to be independent from the storage conditions (i.e. frozen or non-frozen) but was sample-dependent. Some samples showed many deformed particles and/or only virus particles without spikes. Deformation of virus particles and spike visibility in infected cell culture are most probably due to false or incomplete assembly at later stages of infection or virus production in cell culture [30]. It can be expected that at least in some patients the infection already lasted for a few days until the swab sample was taken and then contain mainly virus particles from later cellular infection stages which could reveal deformations and/or less spikes, like the particles in later stages of infected cell cultures. We measured the particle size and spike density of virus particles in two randomly selected swab samples (selection criterion: visible spikes) and compared them with the size and spike density of virus particles of the same virus variant in cell culture. The size of particles was very similar and only slightly elevated by 3–4 nm in swab sample 7, which revealed also a reduced roundness indicating a deformation of the particles. The spike density of the particle profiles was also similar, with a slight tendency to lower spike numbers in the swab samples, which could be due to a reduced visibility, a reduced integration (as discussed above) or a loss of spikes. However, the spike numbers per virus profile indicate that the virus particles in patients possess a similar number of spikes (i.e. 20–25 spikes per virus particle; see [24]) as viruses in cell culture, if the spikes are generally present.

The sedimentation method for swab suspensions presented here is compatible with embedding in epoxy and acrylic resin, including rapid infiltration and polymerization. This allows using the method for rapid diagnostics in cases of emergencies, for instance, the emergence of unknown or unconventional pathogens. However, spike visibility was reduced after osmium tetroxide post-fixation and rapid embedding in the acrylic resin LR White. Block-contrasting with tannic acid and/or uranyl acetate, as performed in the epoxy embedding protocol, could probably help to increase spike visibility, but would increase processing time and limiting the benefit of using LR White. In case of SARS-CoV-2, the reduced spike visibility was not relevant for diagnostics because the viruses could be identified by finding them in intracellular membrane-bound compartments in high numbers which is a typical feature of coronaviruses [25].

A requirement for a fast detection of viruses by thin section EM using the sedimentation method described in this paper is a comparatively high particle concentration of the swab suspension. According to experiments with *Vaccinia virus* suspensions of defined concentration, a particle concentration of 10^7^ particles/ml is necessary to allow an immediate detection of viruses without performing an extensive search of several sections at the microscope. At 10^6^ particles/ml not all of the samples revealed viruses in the first section through the pellet, which would require taking more sections and extended time for microscopy. The SARS-CoV-2-positive swab samples used in this study contained RNA loads far above approx. 10^7^ copies/ml (Ct 20.4 corresponds to 2 × 10^7^ RNA copies/ml; [19]). It was therefore not surprising that we could find coronaviruses in all of the swab samples tested. However, it is still unclear what the relationship is between the number of RNA copies detected and the number of virus particles present. From cultivation assays it is known that already at a viral RNA load of 10^6^ particles per ml the cultivation success drops to 50% of the cultures infected [4] which indicates that at least the fraction of infectious virus is much lower than the RNA load. Using our novel method could help to investigate the reason for this observation and clarify how many virus particles are present and in which condition. A reduced spike availability at the particle surface, as observed in some of the samples investigated here (see above), could be an explanation.

In summary, we presented a method for thin section EM of naso/oropharyngeal swab samples, which allows the detection and analysis of SARS-CoV-2 particles in the extra- and intracellular context of the acute phase of the infection, thereby complementing studies using autopsies, biopsies and cell cultures. The method could be relevant for the study of further virus variants of concern or of other emerging infectious pathogens affecting the upper respiratory tract, because it offers direct and easy access to the pathogens produced by the natural host including infected or affected cells for the study of their pathogenesis. Moreover, it is likely that the method could be extended to other sample types, such as broncho-alveolar lavage or urine.

## Supplementary Information

**Additional file 1: Video S1**. Recovery of the sediment after ultracentrifugation

**Additional file 2: Table S1**. Step-by-step protocol of sediment embedding in Epon

**Additional file 3: Table S2**. Step-by-step protocol of rapid sediment embedding in LR White resin

**Additional file 4:** **Fig. S1**. Transmission EM of a thin section through the sediment of swab sample 2 (Epon embedding).** A** A group of SARS-CoV-2 particles (*rectangle*) is localized within mainly vesicular material and bacteria (*ba*) of various profile shape. At the lower edge of the image a keratinocyte (*kc*) is visible.** B** Enlarged view of the coronavirus particle group marked with a rectangle in frame** A**. Only few virus particles reveal spikes (*arrowheads*) and some particles appear deformed (*arrows*). Scale bar in A = 2 µm, in B = 100 nm.

**Additional file 5: Fig. S2**. Transmission EM of thin sections through the sediment of swab sample 1 (Epon embedding). Single SARS-CoV-2 particles are shown in the vicinity of keratinocytes.** A** A SARS-CoV-2 particle (*rectangle*) is localized between two keratinocytes (*kc*) and a bacterial cell (*ba*). Inset: Magnified view of the virus particle which reveals the typical features of coronaviruses: i.e. an oval shape and a profile size within the 60–140 nm range, a limiting bio-membrane, club-shaped surface spikes and a granular particle interior.** B** The rim of another keratinocyte, which reveals characteristic tonofilaments (*tf*) and a SARS-CoV-2 particle (*arrow*) close to the cell surface. Scale bar in A = 1 µm, Inset = 100 nm, in B = 200 nm.

**Additional file 6: Fig. S3 **Transmission EM of thin sections through the sediment of the PCR-negative swab sample 10 (Epon embedding). **A** Overview image of an observation field which shows a keratinocyte (*kc*) and, in the extracellular space, crystalline (*arrow*) and vesicular (*arrowhead*) structures (the image file is available in data set 6). Examples for smaller vesicular profiles, indicated by the three boxes, are magnified in **B-D**. For reference, virus particle profiles (*arrowheads*) from PCR-positive swab samples are shown in **E-G**. All particle profiles indicated by numbers in **B-D** are in the correct size range of virus particle profiles, but lack other typical features of virus particles: profile 1 lacks the spike proteins and the interior granules are smaller in size and less dense; profile2 lacks a clear membrane envelop, spikes and a granular interior; profiles 3 and 4 lack spikes and a granular interior; profiles 5 lack the typical electron-dense granular interior. Scale bar in A = 2 µm and in B-G = 100 nm.

**Additional file 7: Fig. S4**. Electron tomography of thin sections through the sediment of swab sample 7.** A** A computed slice (approx. 10 nm thick) from a tomogram of four SARS-CoV-2 particles (*1-4*) demonstrates the spike morphology (*arrowheads*) and interior granular substructure (*arrows*) much clearer than by conventional transmission EM of sections at standard section thickness (i.e. 60–70 nm). **B **A computed slice (approx. 6 nm thick) from a tomogram of double-membrane vesicles (*) in a ciliated cell. Note that the inner membrane is not entirely continuous (*arrows*) which indicates instability. The loose filamentous content of the vesicles is typical and most likely represent double-stranded RNA [31]. Scale bar = 200 nm.

**Additional file 8: Fig. S5**. Transmission EM of thin sections through the sediment of swab sample 6 (Epon embedding). Images show comparatively well-preserved non-infected cells.** A** Part of ciliated cell with cross- and longitudinal sections through cilia (*arrows*) and their basal bodies (*arrowheads*). Mitochondria (*mi*) reveal well-preserved membranes but a somewhat extracted matrix.** B** Part of an un-identified cell with dense cytoplasm, short microvilli (*arrowheads*) and well-preserved membranes, including the envelop (*arrows*) of the nucleus (*nu*). Mitochondria (*mi*) and several other membrane-bound compartments, such as the rough endoplasmic reticulum (*er*) are visible. Scale bars = 1 µm.

**Additional file 9: Fig. S6**. Transmission EM of thin sections through the sediment of frozen and non-frozen swab samples (Epon embedding).** A**,** B** Ciliated cells in non-frozen samples** A** show more structural detail than ciliated cells in frozen samples (**B**). In non-frozen cells** A** membranes are at least partially preserved (*arrowheads*) and the cytoplasm reveals various organelles, such as mitochondria (*mi*). Membrane preservation in frozen samples** B** is poor (see naked microtubular backbone of the cilia in** B**; arrows) and the cytoplasm (*) appears extracted.** C**,** D** Keratinocytes in frozen (**C**) and non-frozen** D** samples reveal no obvious differences regarding their structural appearance. The cytoplasm is filled with numerous tonofilaments (*tf*). Scale bar in A, B = 1µm, in C, D = 0.5 µm.

**Additional file 10: Fig. S7**.Transmission EM of a thin section through the sediment of a* Vaccinia virus* suspension (LR White embedding). A few virus particle profiles (*) are embedded in the filamentous matrix of the agarose (*arrowheads*). The inset shows a virus particle profile which reveals the characteristic dumb-bell-shaped inner core of poxviruses. Scale bars = 200 nm.

## Data Availability

Five image data sets recorded from the samples are available at the zenodo repository: (1) Data set 01 (from sample No. 7) https://zenodo.org/record/6653455. (2) Data set 02 (from sample No. 7) https://zenodo.org/record/6653529. Data set 03 (from sample No. 8) https://zenodo.org/record/6653558. Data set 04 (from sample No. 10) https://zenodo.org/record/6653584. Data set 05 (from sample No. 11) https://zenodo.org/record/6653642. Data set 06 (from sample No. 10) https://zenodo.org/record/7143199. Data set 07 (from sample No. 11) https://zenodo.org/record/7143199.

## Abbreviations

COVID19: Coronavirus disease 2019
EM: Electron microscopy
qRT-PCR: Quantitative real-time polymerase-chain reaction
SARS-CoV-2: Severe acute respiratory syndrome virus 2
