# Supplementary material for: Visualization of SARS-CoV-2 particles in naso/oropharyngeal swabs by thin section electron microscopy
Source: Virol J. 2023 Feb 6;20:21. doi: 10.1186/s12985-023-01981-9 (PMC9901382; doi:10.1186/s12985-023-01981-9)
Supplement: Supplementary file 2 — Additional file 2: Table S1. Step-by-step protocol of sediment embedding in Epon [file 12985_2023_1981_MOESM2_ESM.pdf]

**Table S1.** Stepwise protocol for embedding in epoxy resin (Laue 2010)

| Step | Solution/Mixture/Solvent | Duration of incubation [min] | Temperature | Tissue processor |
|---|---|---|---|---|
| 1 | HEPES buffer, 0.05 M | 5 | RT | no |
| 2 | HEPES buffer, 0.05 M | 5 | RT | no |
| 3 | HEPES buffer, 0.05 M | 5 | RT | no |
| 4 | Osmium tetroxide, 1% in water | 60 | RT | no |
| 5 | Distilled water | 5 | RT | no |
| 6 | Distilled water | 5 | RT | no |
| 7 | Distilled water | 5 | RT | no |
| 8 | Tannic acid, 0.1% in 0.05 M HEPES buffer | 30 | RT | yes |
| 9 | $Na_2SO_4$ 1% in 0.05 M HEPES buffer | 10 | RT | yes |
| 10 | $Na_2SO_4$ 1% in 0.05 M HEPES buffer | 10 | RT | yes |
| 11 | Distilled water | 10 | RT | yes |
| 12 | Distilled water | 10 | RT | yes |
| 13 | Distilled water | 10 | RT | yes |
| 14 | Uranyl acetate, 2% in distilled water | 120 | RT | yes |
| 15 | Ethanol, 30% | 30 | RT | yes |
| 16 | Ethanol, 50% | 30 | RT | yes |
| 17 | Ethanol, 70% | 30 | RT | yes |
| 18 | Ethanol, 95% | 60 | RT | yes |
| 19 | Ethanol, abs. | 60 | RT | yes |
| 20 | Ethanol, abs. | 60 | RT | yes |
| 21 | Propylene oxide | 30 | RT | yes |
| 22 | Propylene oxide | 30 | RT | yes |

| | | | | |
|---|---|---|---|---|
| 23 | Propylene oxide / Epon 2+1 | 180 | RT | yes |
| 24 | Propylene oxide / Epon 1+1 | 180 | RT | yes |
| 25 | Propylene oxide / Epon 1+3 | 180 | RT | yes |
| 26 | Epon | 120 | RT | yes |
| 27 | Epon | 180 | RT | yes |
| 28 | Epon | 240 | RT | no |
| 29 | Epon - final embedding in silicone moulds | - | RT | no |
| 30 | Polymerization | 2 days | 60 °C | no |

Tissue processor = Leica EM TP (Leica Microsystems)

**References**

Laue, M. Electron microscopy of viruses. *Method. Cell Biol.* **96,** 1-20 (2010).
