# Supplementary material for: Visualization of SARS-CoV-2 particles in naso/oropharyngeal swabs by thin section electron microscopy
Source: Virol J. 2023 Feb 6;20:21. doi: 10.1186/s12985-023-01981-9 (PMC9901382; doi:10.1186/s12985-023-01981-9)
Supplement: Supplementary file 3 — Additional file 3: Table S2. Step-by-step protocol of rapid sediment embedding in LR White resin [file 12985_2023_1981_MOESM3_ESM.pdf]

Table S2. Stepwise protocol for rapid embedding in LR White resin (Laue 2010)

Step	Solution/Mixture/Solvent	Duration of incubation [min]	Temperature
1	HEPES buffer, 0.05 M	5	RT
2	HEPES buffer, 0.05 M	5	RT
3	Osmium tetroxide, 1% in water	30	RT
5	Distilled water	2	RT
6	Distilled water	2	RT
15	Ethanol, 70%	5	ice
17	Ethanol, 100%	5	ice
18	Ethanol, 100%	5	ice
19	LR White / Ethanol (100%) 1+1	5	ice
20	LR White	5	ice
21	LR White	10	ice
22	LR White with 5 µl accelerator/ml in airfuge vials; transfer of samples for final embedding	-	ice
23	Polymerization	60	ice
24	Polymerization	10	60°

References

Laue, M. Electron microscopy of viruses. *Method. Cell Biol.* **96,** 1-20 (2010).
