# Supplementary figures and images for: Visualization of SARS-CoV-2 particles in naso/oropharyngeal swabs by thin section electron microscopy
Source: Virol J. 2023 Feb 6;20:21. doi: 10.1186/s12985-023-01981-9 (PMC9901382; doi:10.1186/s12985-023-01981-9)

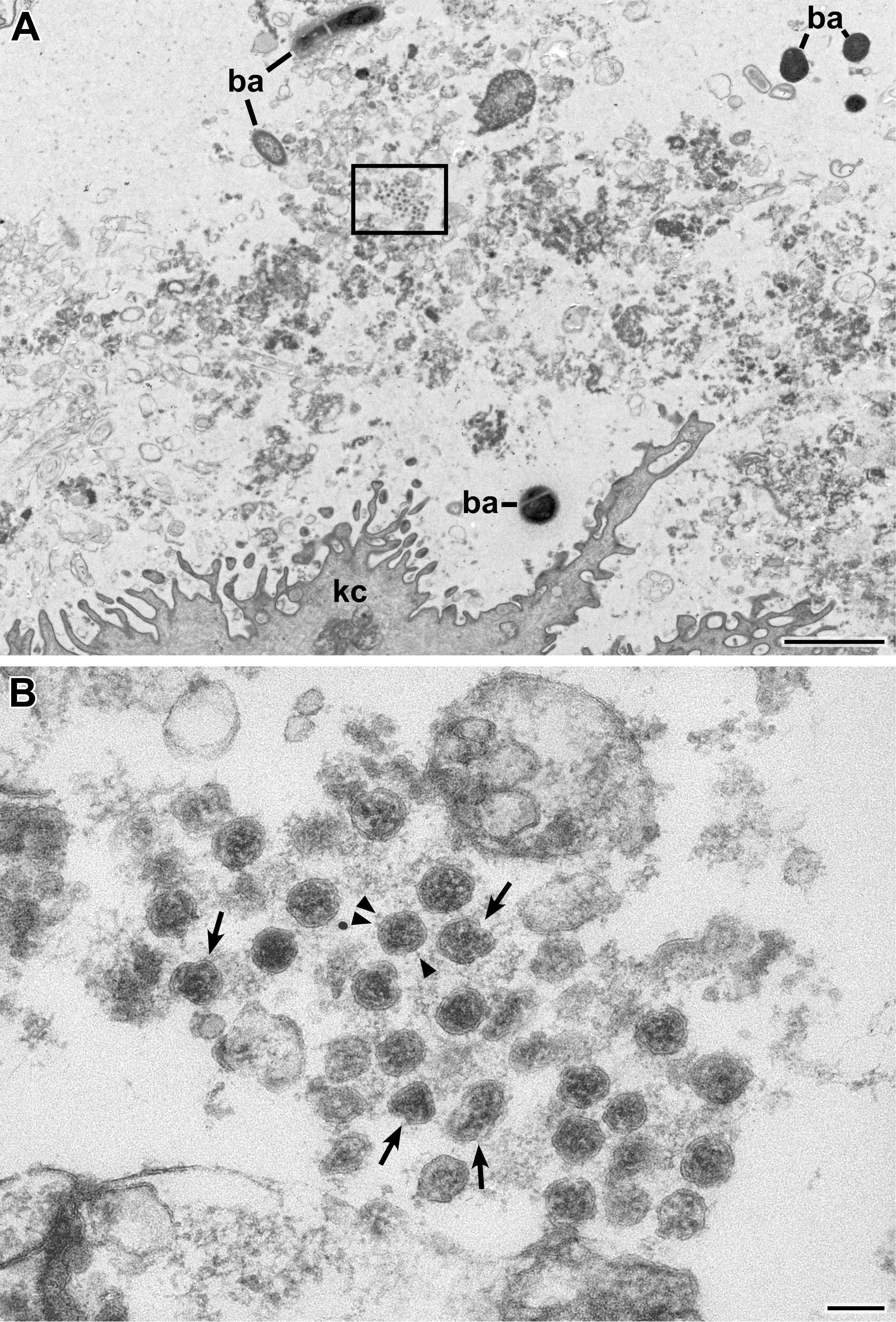

Supplement: Supplementary file 4 — Additional file 4: Fig. S1. Transmission EM of a thin section through the sediment of swab sample 2 (Epon embedding). A A group of SARS-CoV-2 particles (rectangle) is localized within mainly vesicular material and bacteria (ba) of various profile shape. At the lower edge of the image a keratinocyte (kc) is visible. B Enlarged view of the coronavirus particle group marked with a rectangle in frame A. Only few virus particles reveal spikes (arrowheads) and some particles appear deformed (arrows). Scale bar in A = 2 µm, in B = 100 nm. [file 12985_2023_1981_MOESM4_ESM.tif]

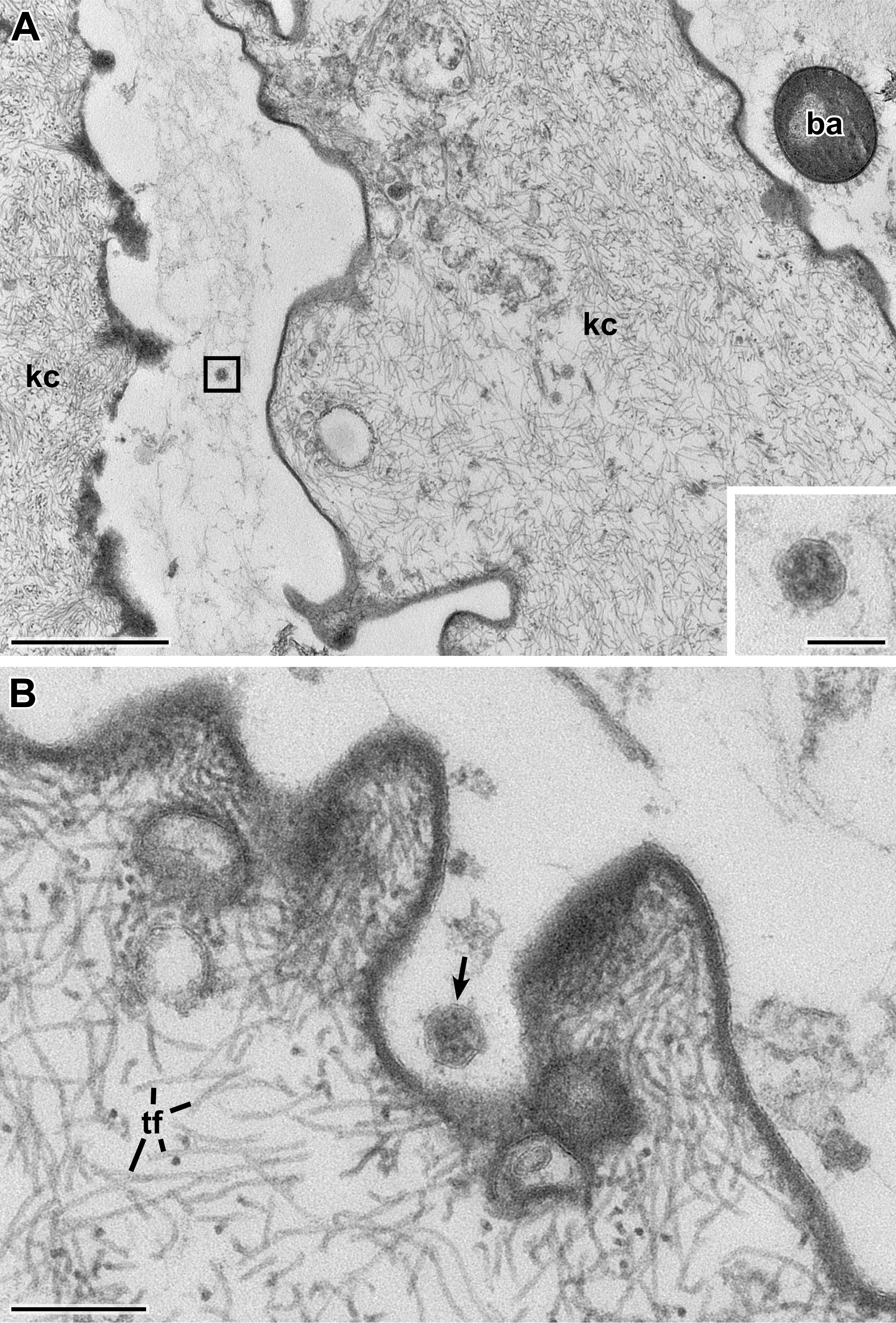

Supplement: Supplementary file 5 — Additional file 5: Fig. S2. Transmission EM of thin sections through the sediment of swab sample 1 (Epon embedding). Single SARS-CoV-2 particles are shown in the vicinity of keratinocytes. A A SARS-CoV-2 particle (rectangle) is localized between two keratinocytes (kc) and a bacterial cell (ba). Inset: Magnified view of the virus particle which reveals the typical features of coronaviruses: i.e. an oval shape and a profile size within the 60–140 nm range, a limiting bio-membrane, club-shaped surface spikes and a granular particle interior. B The rim of another keratinocyte, which reveals characteristic tonofilaments (tf) and a SARS-CoV-2 particle (arrow) close to the cell surface. Scale bar in A = 1 µm, Inset = 100 nm, in B = 200 nm. [file 12985_2023_1981_MOESM5_ESM.tif]

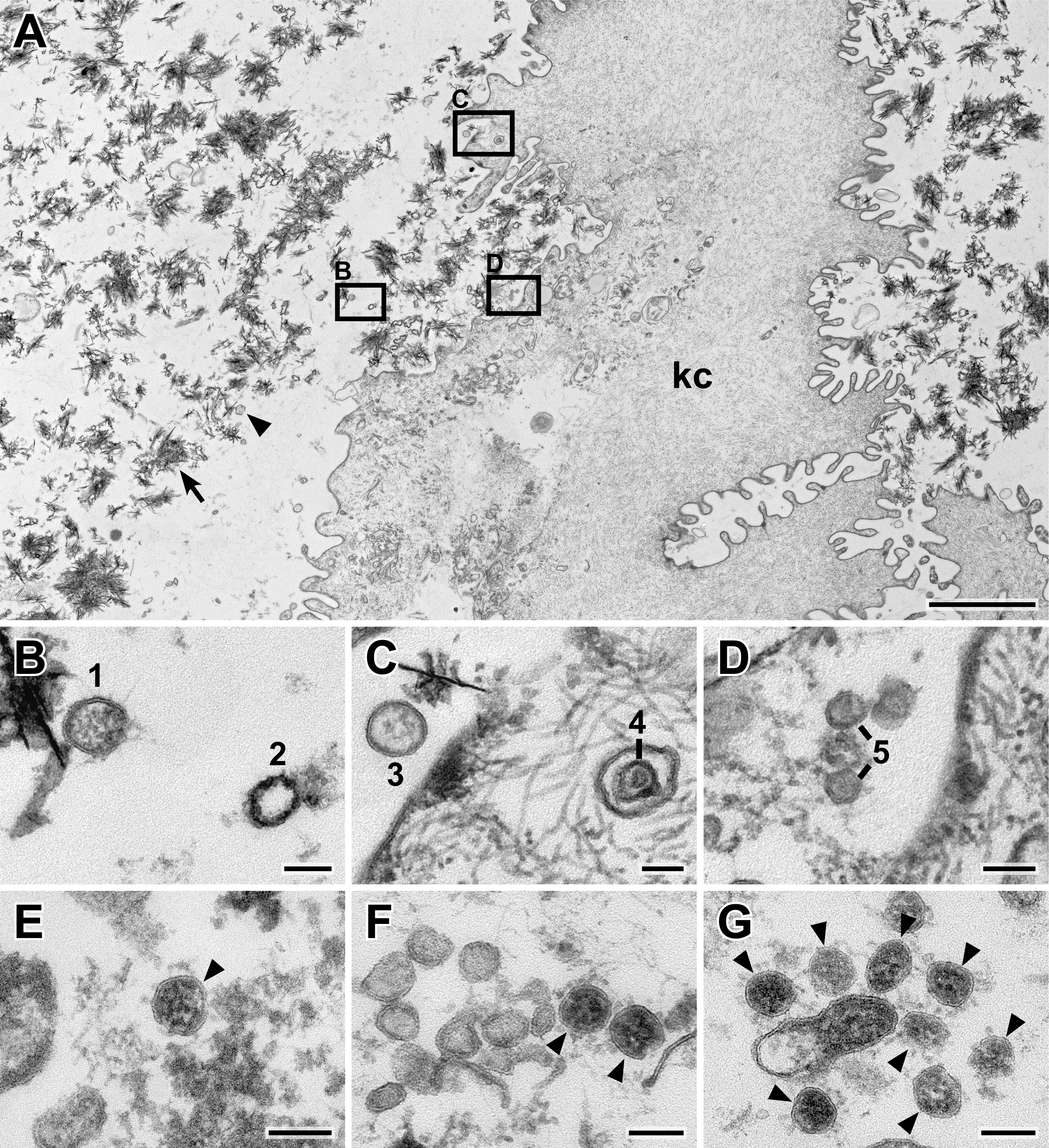

Supplement: Supplementary file 6 — Additional file 6: Fig. S3 Transmission EM of thin sections through the sediment of the PCR-negative swab sample 10 (Epon embedding). A Overview image of an observation field which shows a keratinocyte (kc) and, in the extracellular space, crystalline (arrow) and vesicular (arrowhead) structures (the image file is available in data set 6). Examples for smaller vesicular profiles, indicated by the three boxes, are magnified in B-D. For reference, virus particle profiles (arrowheads) from PCR-positive swab samples are shown in E-G. All particle profiles indicated by numbers in B-D are in the correct size range of virus particle profiles, but lack other typical features of virus particles: profile 1 lacks the spike proteins and the interior granules are smaller in size and less dense; profile2 lacks a clear membrane envelop, spikes and a granular interior; profiles 3 and 4 lack spikes and a granular interior; profiles 5 lack the typical electron-dense granular interior. Scale bar in A = 2 µm and in B-G = 100 nm. [file 12985_2023_1981_MOESM6_ESM.tif]

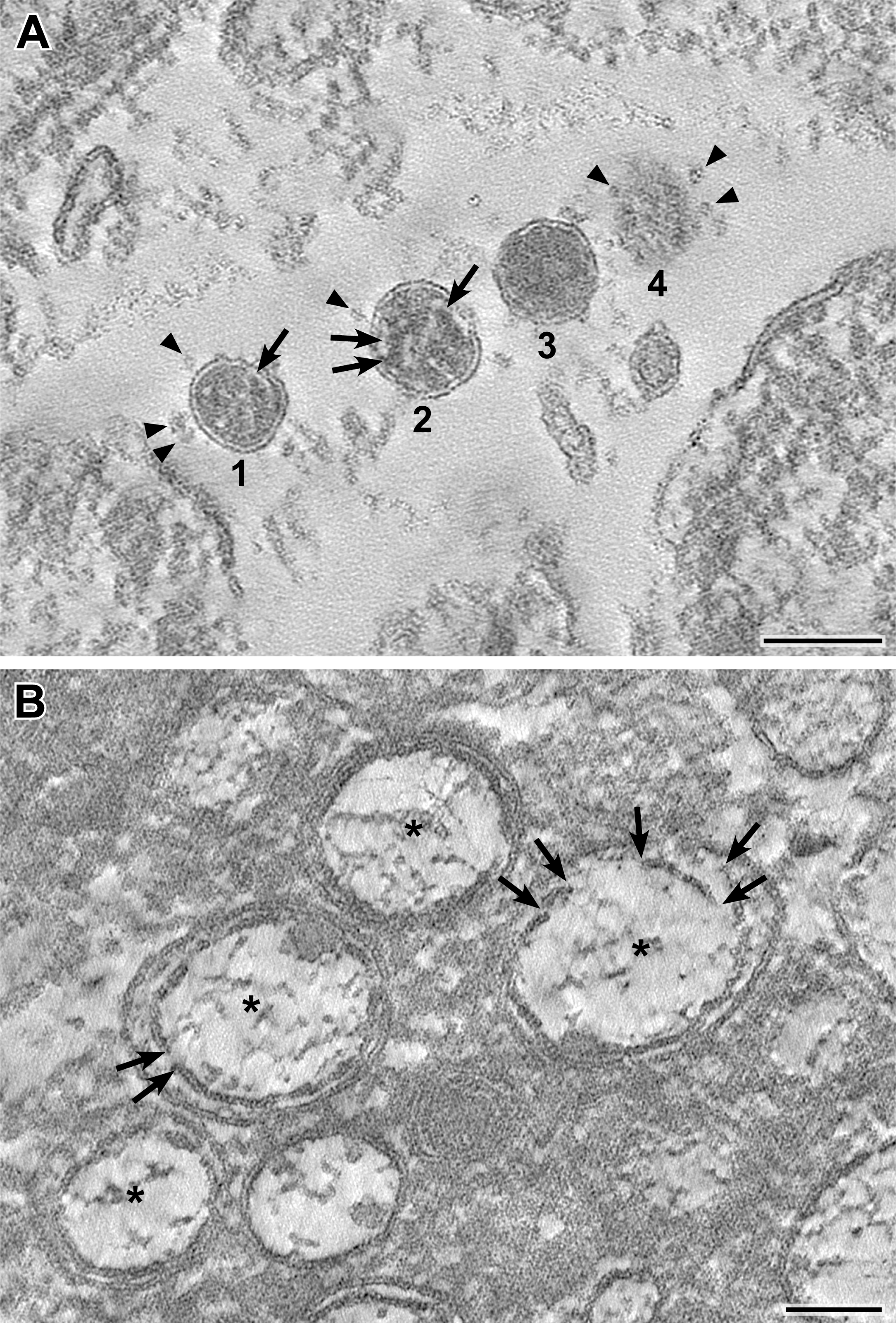

Supplement: Supplementary file 7 — Additional file 7: Fig. S4. Electron tomography of thin sections through the sediment of swab sample 7. A A computed slice (approx. 10 nm thick) from a tomogram of four SARS-CoV-2 particles (1-4) demonstrates the spike morphology (arrowheads) and interior granular substructure (arrows) much clearer than by conventional transmission EM of sections at standard section thickness (i.e. 60–70 nm). B A computed slice (approx. 6 nm thick) from a tomogram of double-membrane vesicles (*) in a ciliated cell. Note that the inner membrane is not entirely continuous (arrows) which indicates instability. The loose filamentous content of the vesicles is typical and most likely represent double-stranded RNA [31]. Scale bar = 200 nm. [file 12985_2023_1981_MOESM7_ESM.tif]

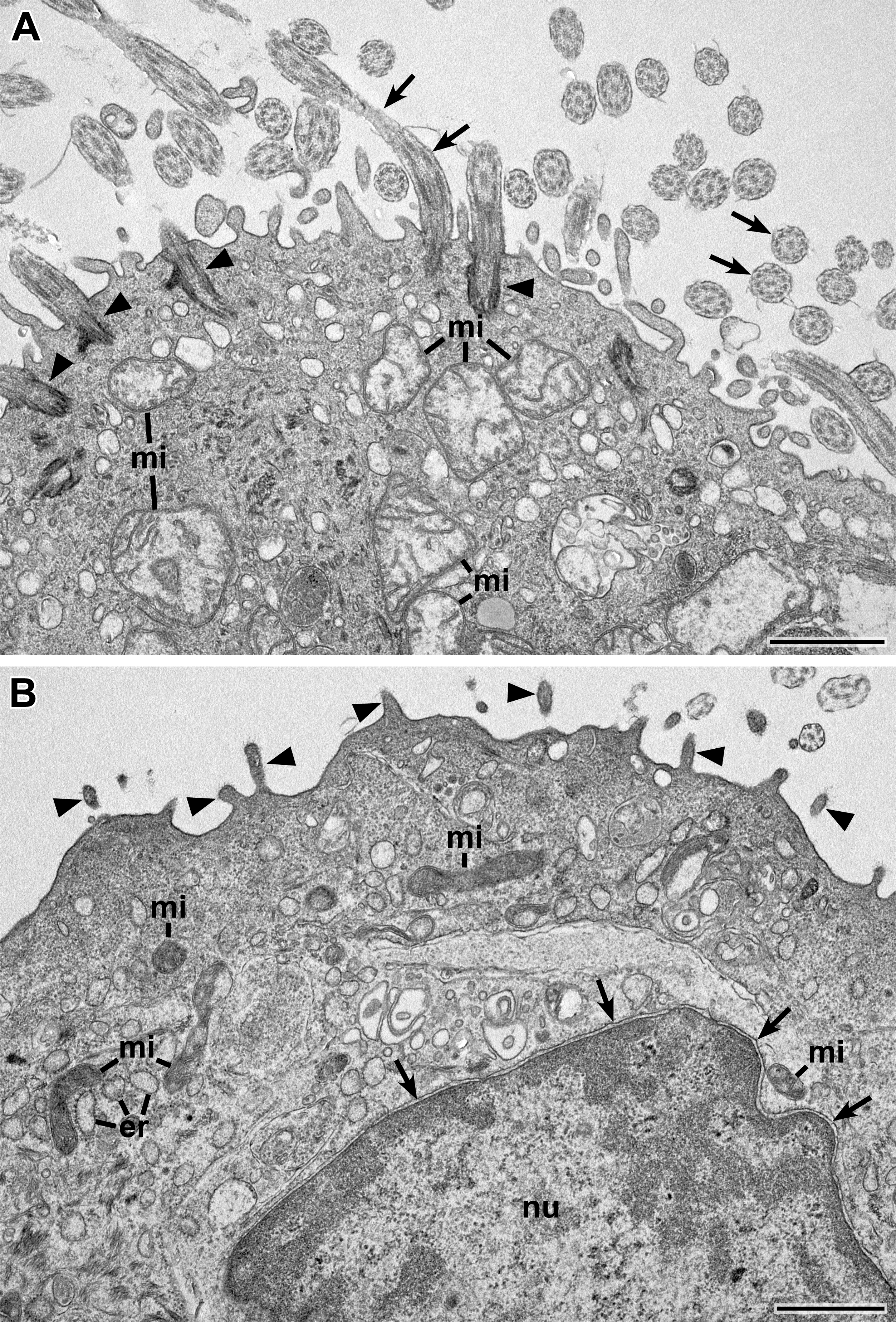

Supplement: Supplementary file 8 — Additional file 8: Fig. S5. Transmission EM of thin sections through the sediment of swab sample 6 (Epon embedding). Images show comparatively well-preserved non-infected cells. A Part of ciliated cell with cross- and longitudinal sections through cilia (arrows) and their basal bodies (arrowheads). Mitochondria (mi) reveal well-preserved membranes but a somewhat extracted matrix. B Part of an un-identified cell with dense cytoplasm, short microvilli (arrowheads) and well-preserved membranes, including the envelop (arrows) of the nucleus (nu). Mitochondria (mi) and several other membrane-bound compartments, such as the rough endoplasmic reticulum (er) are visible. Scale bars = 1 µm. [file 12985_2023_1981_MOESM8_ESM.tif]

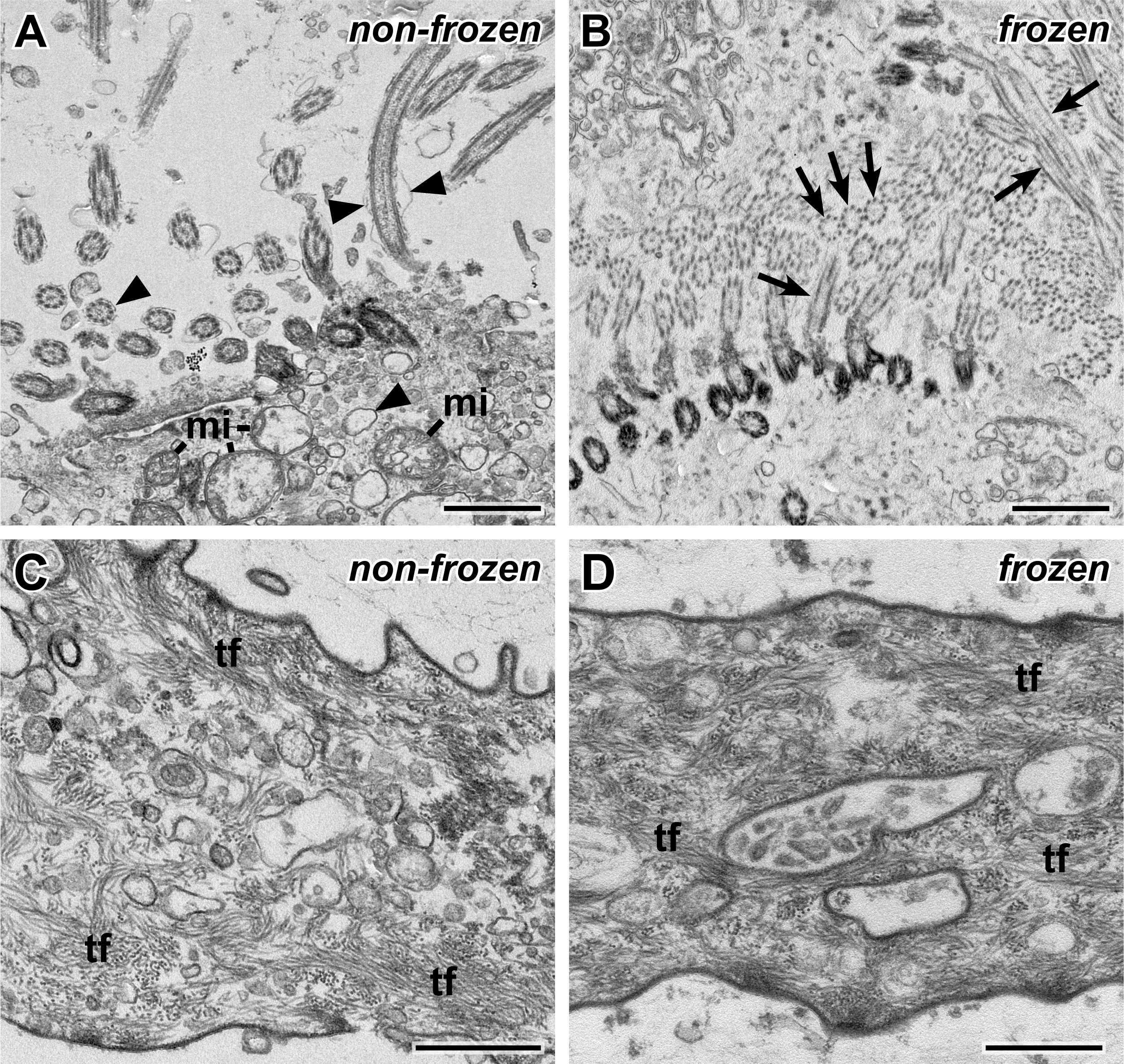

Supplement: Supplementary file 9 — Additional file 9: Fig. S6. Transmission EM of thin sections through the sediment of frozen and non-frozen swab samples (Epon embedding). A, B Ciliated cells in non-frozen samples A show more structural detail than ciliated cells in frozen samples (B). In non-frozen cells A membranes are at least partially preserved (arrowheads) and the cytoplasm reveals various organelles, such as mitochondria (mi). Membrane preservation in frozen samples B is poor (see naked microtubular backbone of the cilia in B; arrows) and the cytoplasm (*) appears extracted. C, D Keratinocytes in frozen (C) and non-frozen D samples reveal no obvious differences regarding their structural appearance. The cytoplasm is filled with numerous tonofilaments (tf). Scale bar in A, B = 1µm, in C, D = 0.5 µm. [file 12985_2023_1981_MOESM9_ESM.tif]

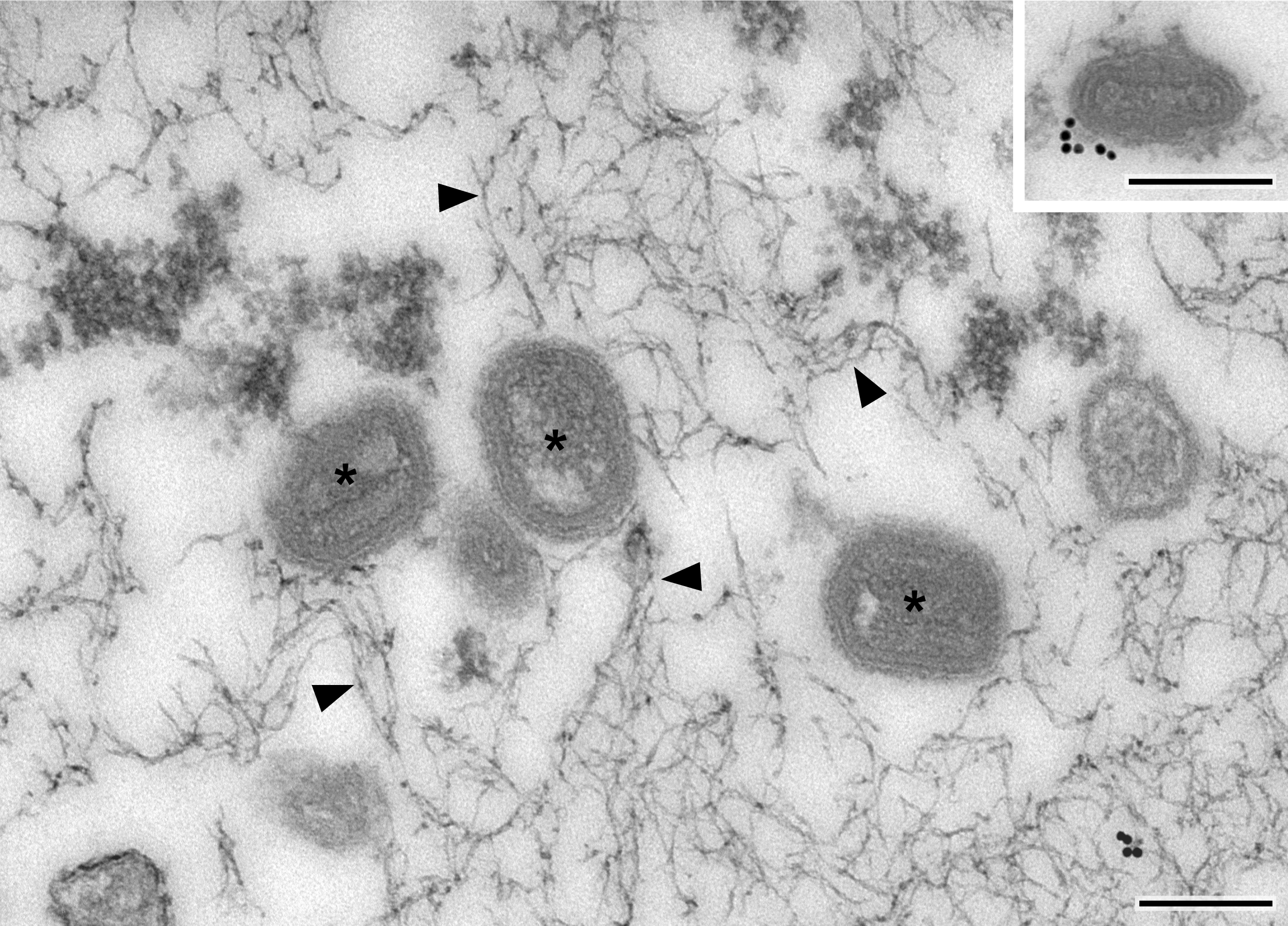

Supplement: Supplementary file 10 — Additional file 10: Fig. S7.Transmission EM of a thin section through the sediment of a Vaccinia virus suspension (LR White embedding). A few virus particle profiles (*) are embedded in the filamentous matrix of the agarose (arrowheads). The inset shows a virus particle profile which reveals the characteristic dumb-bell-shaped inner core of poxviruses. Scale bars = 200 nm. [file 12985_2023_1981_MOESM10_ESM.tif]
